# Biopsy-based optimization and calibration of a signal-intensity-ratio-based MRI method (1.5 Tesla) in a dextran-iron loaded mini-pig model, enabling estimation of very high liver iron concentrations

**DOI:** 10.1007/s10334-021-00998-x

**Published:** 2022-01-17

**Authors:** Peter D. Jensen, Asbjørn H. Nielsen, Carsten W. Simonsen, Kenneth K. Jensen, Martin Bøgsted, Anne B. H. Jensen, Benedict Kjaergaard

**Affiliations:** 1 Department of Hematology, University Hospital, Aalborg, Denmark; 2 Department of the Built Environment, Aalborg University, Aalborg, Denmark; 3 Department of Radiology, Imaging Research Unit, Aalborg University Hospital, Aalborg, Denmark; 4 Department of Clinical Medicine, Aalborg University, Aalborg, Denmark; 5 The Faculty of Medicine, Aalborg University, Aalborg, Denmark; 6 Biomedical Research Laboratory, Aalborg University Hospital, Aalborg, Denmark; 7 Department of Cardiothoracic Surgery, Aalborg University Hospital, Aalborg, Denmark

**Keywords:** Magnetic resonance imaging, Quantitative imaging, Iron overload, Liver iron concentration, Experimental animal models

## Abstract

**Objective:**

Magnetic resonance imaging (MRI)-based techniques for non-invasive assessing liver iron concentration (LIC) in patients with iron overload have a limited upper measuring range around 35 mg/g dry weight, caused by signal loss from accelerated T1-, T2-, T2* shortening with increasing LIC. Expansion of this range is necessary to allow evaluation of patients with very high LIC.

**Aim:**

To assess measuring range of a gradient-echo R2* method and a T1-weighted spin-echo (SE), signal intensity ratio (SIR)-based method (TE = 25 ms, TR = 560 ms), and to extend the upper measuring range of the SIR method by optimizing echo time (TE) and repetition time (TR) in iron-loaded minipigs.

**Methods:**

Thirteen mini pigs were followed up during dextran-iron loading with repeated percutaneous liver biopsies for chemical LIC measurement and MRIs for parallel non-invasive estimation of LIC (81 examinations) using different TEs and TRs.

**Results:**

SIR and R2* method had similar upper measuring range around 34 mg/g and similar method agreement. Using TE = 12 ms and TR = 1200 ms extended the upper measuring range to 115 mg/g and yielded good method of agreement.

**Discussion:**

The wider measuring range is likely caused by lesser sensitivity of the SE sequence to iron, due to shorter TE, leading to later signal loss at high LIC, allowing evaluation of most severe hepatic iron overload. Validation in iron-loaded patients is necessary.

## Introduction

Iron overload may develop in conditions with abnormally increased intestinal iron absorption, as in patients with hereditary hemochromatosis and patients with iron-loading anemias (e.g. ß-thalassemia) and can be caused by chronic treatment with blood transfusions or both [1]. In hereditary hemochromatosis, the excess iron is primarily stored within the liver [2], while in patients with transfusional iron overload, the liver accounts for approximately 70 to 80% of the total body iron stores [3], while the rest is primarily deposited within the spleen [3]. The excess iron is sequestered as ferritin particles and hemosiderin aggregates [4]. During progressive iron accumulation, the body’s capacity for safe sequestration of excess iron may be overwhelmed [5], eventually leading to hepatocellular injury [6], liver fibrosis or even cirrhosis [7–9], cardiomyopathy, and heart failure [10–13], endocrinal dysfunction [14], and reduced long-term survival [10, 12]. In ß-thalassemia and other iron-loading anemias, the risk of these complications is shown to be associated with the liver iron concentration (LIC): LIC exceeding 17 mg/g dry weight are associated with hepatocellular damage [15] and increased risk for cardiac iron overload [16–19].

The current gold standard to quantify the total body iron burden is by chemical measurement of the LIC in a percutaneous liver biopsy [20, 21]. This is a consequence of the fact that the liver represents the dominant iron storage organ in the body [22], and the existing evidence for a close, direct relationship between the total body iron and the LIC [16, 20, 23]. Repeated LIC measurements enable monitoring changes in total body iron burden in patients with transfusional iron overload on iron chelation therapying to evaluate the effectiveness of the therapy [24, 25].

Today, non-invasive techniques are widely used for the estimation of LIC instead of the chemical measurement in a percutaneous liver biopsy because of the risk of bleeding complications [26–28]. Recent guidelines advocate estimating LIC noninvasively by magnetic resonance imaging (MRI) to identify patients with iron overload and to guide titration of chelation therapy [29–31].

The basis for using MRI for non-invasive estimation of the LIC is that ferritin and hemosiderin iron accelerate T1 relaxation, T2 relaxation, and T2* signal decay, causing signal loss on especially on T2-weighted spin-echo (SE) and T2*-weighted gradient echo (GRE) MR images [32]. Close relationships have been demonstrated between these relaxation parameters and LIC [33–42], which likely have promoted the widespread use of MRI for non-invasive estimation of LIC [35–40, 42–52].

Non-invasive estimation of LIC can also be performed by superconducting magnetic susceptometry [53–55]. This method is based on the change in the magnetic susceptibility of the liver in the presence of iron [32]. The measured magnetic field variation is directly related to the amount of tissue iron [56]. This is different in MRI: MRI does not image the iron directly. Instead, it detects the effect of iron on water protons in the investigated liver tissue [32]. Susceptometers are only accessible at a limited number of centers [53, 54, 57], while MRI scanners are widely available today. This may partly explain why MRI is the primary approach today for the non-invasive estimation of LIC [45].

In general, there are two MRI-based strategies for estimation of LIC, by relaxometry, based on measurement of absolute R2 [36, 42], or R2* [35–37, 39, 40, 44, 46] and by methods measuring the signal-intensity ratio (SIR) between liver and skeletal muscle, acting as a reference tissue [43, 47], assessed either from SE [38, 47–52] or GRE sequences [38, 39, 43].

The methods differ among other things regarding prevalence in use, extent of calibration, and measuring range: The R2* method is in most widespread use in clinical practice, likely because it is a faster and easier method than the R2 method [42].

The most widely used SIR method is that of Gandon et al. [43], which is based on GRE sequences. In earlier studies, SE sequences have been used for the SIR method [38, 47–52]. We have for many years used a SIR method at our Department of Radiology, based on a T1-weighted SE sequence [47], for non-invasive estimation of the LIC in clinical settings and research in iron overload [47, 48]. The method uses an echo time (TE) of 25 ms, a consequence of scanner limitations when the method was calibrated. As far as we know, the method is not in clinical use at other centers.

The best-calibrated method in use is an R2 method, known as FerriScan® [42]. The method uses a multi-slice single-spin echo (SE) pulse sequence [25]. Its calibration is based on liver biopsies from 105 patients (23 with hereditary hemochromatosis, 50 patients with thalassemia (nine patients had been treated with regular blood transfusion and iron chelation therapy), and 32 patients had hepatitis) [42].

The best-calibrated R2* method is based on a breath-hold single GRE pulse sequence [36]. Its calibration was based on biopsies from 22 patients (9 patients had thalassemia major and 2 had intermedia, 10 had sickle cell disease and one had Blackfan-Diamond syndrome) [36]. Only the FerriScan^®^ method has been approved as a clinical device for the assessment of liver iron by regulatory authorities in the USA, Canada, Europe, and Australia [58].

The method of Gandon et al. [43] uses five breath-hold GRE pulse sequences, obtained by adjusting the flip angle or the echo time (TE) to modulate image weighting, to generate T1-weighted, proton-density-weighted, or mild, moderately, or heavily T2*-weighted images. The calibration is based on 139 patients, who were scheduled to undergo liver biopsy for either suspicion of hepatic iron overload or management of chronic hepatitis [43].

The calibration of our method [47] is based on biopsies from eight patients (five patients had hereditary hemochromatosis, two had transfusion-dependent myelodysplastic syndrome, and one had hepatitis) [47].

All methods have a limited upper measuring range. The upper limit of the SIR method is around 30 to 35 mg/g. This is because, in our hands, at these LIC levels, the signal intensity of liver tissue (SI L) approaches background noise. Although the method covers the main part of the patients with iron overload referred to our department for estimation of LIC, we encounter patients with higher LIC levels and cannot evaluate them properly. The upper limit of the measuring range of the SIR method of Gandon et al. [43] is around 20 mg/g and relaxation measurements of R2* are capable to estimate LIC levels up to 35–40 mg/g, depending on the scanner type [24]. The upper limit of the R2 method is not known, but LIC measurements up to 43 mg/g have been published [42].

The objective of the present study was as follows:

To compare our SIR method with an R2* method.

To investigate if the upper limit of the measuring range of our SIR method might be extended by an optimization of the underlying SE sequence. The idea was that the SI L will decay later at increasing LIC by reducing TE from 25 to 12 ms.

To investigate the effect of six different repetition times (TR) on the agreement between MRI measures of LIC and biopsy LIC.

To study the usefulness of the skeletal muscle as a tissue reference.

We investigated these issues in a mini-pig model of iron overload, induced by dextran-iron loading, because this model enables estimation of MRI measures of LIC and parallel measurement of biopsy measures of LIC, by providing easy access to repeated percutaneous liver biopsies.

## Material and methods

### Animals, animal care, and iron-loading:

This study includes data from 14 female Göttingen mini pigs, obtained from the barrier unit at Ellegaard Göttingen mini-pigs A/S, Dalmose, Denmark. Animal facilities, principles of laboratory and animal care were as described earlier [59] and conformed to the requirements of the Danish Animal Experiments Inspectorate (Stationsparken 31–33, DK-2600 Glostrup, Denmark). The mini pigs were from 6 to 8 months of age when included in the study. They were followed up with 1- to 3-month intervals for up to 21 months with transcutaneous liver biopsies and parallel MRI for assessment of hepatic T2*, hepatic SIR, and myocardial T2*. The myocardial T2* data have already been published recently [60]. MRI of the liver was always performed around an hour before the liver biopsy.

**Fig. 1:**
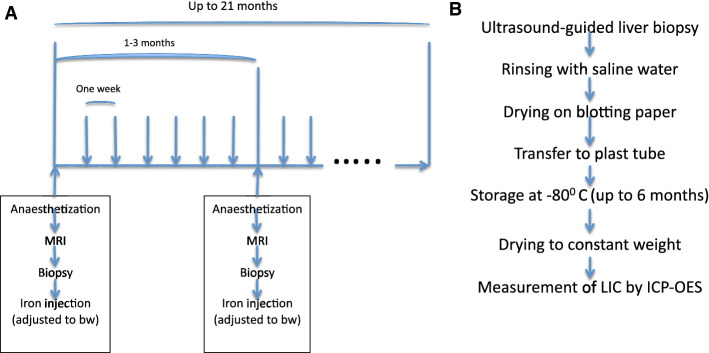
Flow diagram of iron loading and examinations of the mini pigs. **A** gives a timeline for iron loading by weekly dextran-iron injections (vertical arrows) and repeated biopsy/MRI sessions. **B** illustrates the procession of the liver biopsies for the assessment of LIC. BW is the bodyweight of the pig

Iron loading was performed by weekly, intramuscular injections with dextran iron (Uniferon^®^, 200 mg Fe/ml, Pharmacosmos A/S, Roervangsvej 30, 4300 Holbæk, Denmark, product number 474508), as used earlier in rabbits and rats for induction of iron overload [61]. The dose, recommended by the manufacturer, for 1-3-day-old piglets is one ml (200 mg Fe) once (equaling about 139 mg Fe/kg body weight). The applied dose in the present study was from 5 to 140 mg Fe/kg/week. The dextran-iron injections were admistered into the buttocks. A flow diagram illustrates the principles of iron loading by injections and its timely relation to the examinations of the mini pigs (Fig. 1A). Table 1 gives the dose and the duration of iron loading for each pig.

**Table 1:** Details on iron-loading with dextran iron of 14 mini pigs

Pig no	Dose (mg Fe/kg/week)	Months
1	125	15
2	5	21
3	10	21
4	died after 1. biopsy	
5	20	13.5
6	30	2.5
7	35	13
8	140	10.5
9	7.5	13.5
10	10	13.5
11	50	14
12	100	14
13 control	0	0.5
14 control	0	0.5

In pig 2 and 3 the dose was increased to 35 mg/kg/week after 12 months of iron loading

**Fig. 2:**
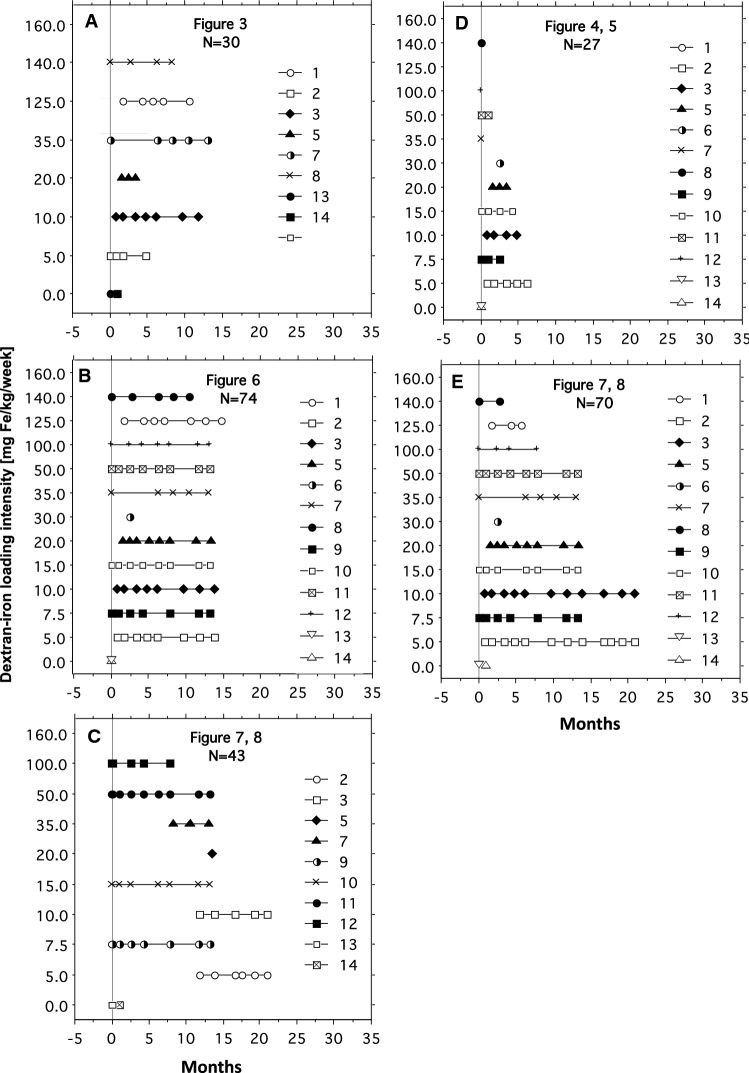
Individual pig timelines of dextran-iron loading and time points of investigations. Each panel illustrates for each figure, presented in the *Results*, the number of investigated pigs, and for each pig (identified by numbers) the dosing of dextran iron in mg Fe/kg/week and the duration of iron loading (months) at each time point of MRI/biopsy during follow-up. In Figs. 7 and 8, not all pigs had a scan at TR = 300 ms. Pigs that had this scan, are illustrated in **E**. For further details see *Materials and Methods*

Pig 4 died during the first MRI/biopsy session due to bleeding complications after the liver biopsy. The data of this pig were excluded from the study, leaving 13 pigs. Dextran-iron injections were always given immediately after the liver biopsy had been taken. At the end of the study, 81 MRI scans with parallel biopsy LIC measurements were available for image analysis. Figure 2 illustrates for each figure, presented in the *Results*, the number of investigated pigs, and for each pig the dosing of dextran iron (mg Fe/kg/week) and duration of iron loading at each time point of parallel MRI/biopsy investigation during follow-up. Due to limited housing capacity, MRI/biopsy sessions were performed at different time points during the iron loading and follow-up, explaining the individual timelines of the pigs.

### Anaesthetization of the animals:

Before each intramuscular injection of dextran iron, the animals were anesthetized with a single dose of Zoletil^®^ 50 Vet (50 mg/ml), containing 8.3 mg/ml tiletamine hydrochloride, 8.3 mg/ml zolazepam hydrochloride, 8.3 mg/ml xylacin, 8.3 mg/ml ketaminol, and 1.7 mg butorphanol (Virbac, 1ère avenue 2065 m LID-06516 Carros, France, product number 568527). The dose was 10 mg/kg body weight, according to the manufacturers' recommendation for dogs (7–10 mg/kg body weight), as described earlier [62]. Before MRI scan, and invasive procedures with biopsies, the pigs were anesthetized with repeated doses of Zoletil^®^ 50 Vet. The trachea was intubated with a 6.5 mm cuffed endotracheal tube. Before breath-hold, during the T2* investigation, general anesthesia was achieved by the addition of 1.2% sevoflurane (Sevoflurane^®^, 1 ml/ml, Baxter, Soeborg, DK, product number 023608). A dedicated MRI-compatible respirator (Servo ventilator 900c, Siemens, Germany) maintained artificial ventilation (N_2_0:O_2_ mixture was 2:1) between breath holds during MRI [63].

### Sampling and procession of liver biopsies:

A schematic of the procedures is given in Fig. 1B. Ultrasound-guided [64] percutaneous liver biopsies were taken. Biopsies were taken by use of biopsy needle Bard^®^ Biopsy-Cut^®^ (14-gauge × 160 mm, Bard, Covington, Georgia, USA, product number 451416). Biopsies were immediately transferred to a 1.5-ml Eppendorf tube^®^ (Eppendorf AG, Hamburg, Germany, product number 0030 120.086) containing 0.9% isotonic sodium chloride solution for rinsing for blood. Afterward, the biopsy was placed on blotting paper (Whatman^®^ filter paper, Merck KGaA Darmstadt, Germany, product number WHA10340810) for about a minute and thereafter transferred to another dry 1.5 ml Eppendorf tube^®^ for storage at − 80 °C.

### Chemical assessment of hepatic tissue iron concentration

The LIC is given as mg/g dry liver tissue. Before measuring the LIC, the biopsy was dried at 95 C for 3 days to constant weight. Immediately thereafter, the biopsy was weighted. The liver sample weight was between 0.36 and 4.5 mg (mean ± Std: 2.52 ± 1.11 mg in 81 samples). A detailed description of the subsequent microwave-assisted acid digestion of the samples, followed by elemental analysis of the digested samples, using inductively coupled plasma-optical emission spectroscopy (ICP-OES) has been given earlier [59].

### MRI for assessment of hepatic SIR and T2*

All MRI scans were performed on a clinical General Electric (GE) 1.5 T Discovery 450 (GE Healthcare, Chicago, IL, USA) at the Imaging Research Unit, Department of Radiology, Aalborg University Hospital, Denmark. The software package of the scanner was version 24. For measurement of the SI L/SI M-ratio, a T1-weighted standard respiratory-compensated SE sequence (GE Healthcare, Chicago, IL, USA) was used. The coil configuration was a body coil. Examinations were performed with the pig in a supine position. Four axial slices through the center of the liver and the paraspinal muscles were imaged. Scanning parameters are given in Table 2.

**Table 2:** Magnetic resonance imaging parameters

	TR (ms)	TE (ms)	Flip angle	Matrix	Bandwith (khz)	FOV (cm)	Slice/space (mm)
Sequence							
GRE	10	1.0, 2.2, 3.5, 4.7, 6.0, 7.2, 8.5, 9.7 10.9, 12.2, 13.4, 14.7, 15.9, 17.2	10	128 × 128	83.3	38	8/1.5
		At high LIC*: 0.9, 1.2, 1.6, 1.9, 2.3, 2.6, 2.9, 3.3, 3.6, 4.0, 4.3, 4.7, 5.0, 5.3, 5.7					
SE	300, 400, 500, 600, 800, 1000, 1200	12 and 25	–	160 × 160	20.38	40	8/3

*FOV* field of view

*Set of TRs used when the SI L approached background levels when scanning livers with high LIC

For measurement of T2*, a spoiled fast-gradient single-echo sequence (GE Healthcare, Chicago, IL, USA) was used. Coil configuration was GE 8 channel HD cardiac phased array (GE Healthcare, Chicago, IL, USA). The coil was placed straight on the center of the MR table and the center of the coil was at the level of the xiphoid process. The pig was in a supine position. Slice orientation was as used for the assessment of SIR of liver tissue. Scanning parameters are given in Table 2.

### Image analysis

**Fig. 3:**
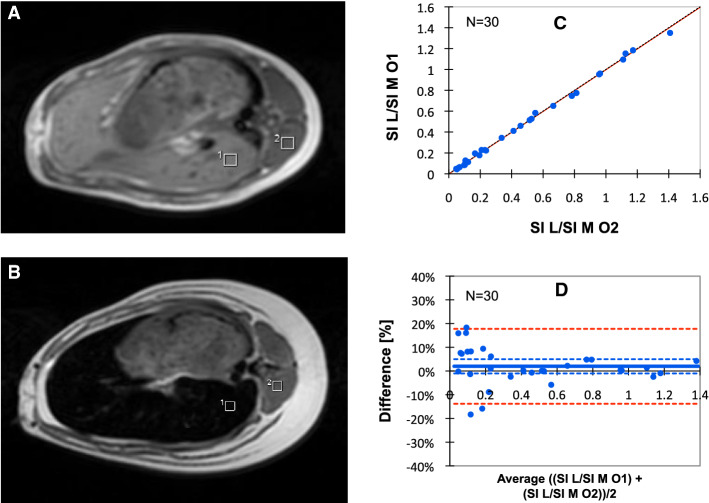
SIR method for measurement of the SI L/SI M-ratio and its inter-observer reproducibility. **A**, **B** show a transverse slice through the center of the liver and the paraspinal muscle of an anesthetized mini pig (no.12) before and after loading with dextran iron (100 mg Fe/kg/week for 14 months). Rectangles represent ROIs for the measurement of mean SI L and mean SI M. **C** The inter-observer reproducibility and agreement between observers 1 and 2, given with the line of identity. **D** Inter-observer reproducibility investigated by Bland–Altman analysis (same data as **C**). SI L/SI M-ratio percentage differences are plotted against the mean SI L/SI M-ratios, assessed by the two observers. The full line represents bias with 95% CI (broken, blue lines). LoAs are indicated by broken, red lines

Measurement of the mean SI L and the mean signal intensity of muscle tissue (SI M) for calculation of the SI L/SI M-ratio was performed in a slice that simultaneously visualized the liver and the paraspinal muscle (m. erector spinae). SI measurements were derived from a region of interest (ROI) of 10 × 10 mm of size, placed in an area of homogeneous liver tissue, avoiding vessels and other sources of artifacts, as illustrated in Fig. 3A, B.

For calculation of hepatic T2*, the StarMap analysis tool (GE Healthcare, Chicago, IL, USA) was used. If the data plot of SI L against TE showed a plateau in the later echo time images, a truncation method was used [65]. Image analysis was performed by an experienced observer, who was blinded to the results of the biopsy LIC measurements.

When investigating the inter-observer reproducibility of SI L/SI M-ratio measurements (Fig. 3C, D), both observers were also blinded to each other. The reproducibility was assessed in 30 scans. They were selected from the 81 available MRI examinations, having parallel biopsy LIC measurements available, in a way that ensured coverage of the LIC range of interest (1 to 135 mg/g).

Figure 3D shows the Bland–Altman analysis of inter-observer SI L/SI M-ratio percentage differences plotted against the mean SI L/SI M-ratio for the two observers. The bias was 1.99% with a 95% confidence interval (CI) from − 1.03 to 8.07%. The limits of agreement (LoAs) were between − 13.82 and 17.79%. The disagreement was largest at SI L/SI M < 0.2, reflecting LIC values of more than 115 mg/g. When excluding these LIC values, a bias of 0.24% was found with a 95% CI from − 1.61 to 2.09% and with LoAs between − 7.04 and 7.52%.

### Human calibration curve

When investigating the transferability of the calibration curve, obtained from the pig model, to patients, we used the human calibration, still in use at our Department of Radiology for our SIR method [47]. The calibration curve is implemented in an in-house software package. The package also enables correction of the SI L/SI M ratio for the use with different TRs during image acquisition, with no correction, if TR = 684 ms. To adjust the SI L/SI M-ratios for the difference in TR, we used the following correction functions, implemented in the package [47]:

1 $$\left( {{\text{SI L}}/{\text{SI M}}} \right)_{{{\text{adjusted}}}} \, = \,{\text{SI L}}/{\text{SI M }} \times {\text{ TR}}\, + \,\alpha \left( {{\text{T}}_{0} - {\text{TR}}} \right)$$

where* T*_0_ is TR = 684 ms. α is approximated by a fourth-degree polynomial, which describes the relation between SI L/SI M (x) and TR [47]as follows:

2 $$\alpha \, = \, - {1}.{\text{98e}} - {2}\, + \,{3}.{\text{77e}} - {4}x{-}{2}.{4}0{\text{e}} - {6}x^{{2}} \, + \,{4}.{\text{82e}} - {9}x^{{3}} {-}{2}.00{\text{e}} - {12}x^{{4}}$$

### Statistics

Upper limits of normal were calculated as mean + (2 × SD). Inter-observer agreement was assessed by the Bland–Altman method [66]. Bias, upper and lower 95% LoAs between biopsy LIC values and LIC estimates, predicted from SI L/SI M-ratio-based calibration curves, were also assessed by the Bland–Altman method.

Exponential model fit of biopsy LIC to SI L or SI L/SI M-ratio was investigated by exponential least-square regression analysis. As data were not normally distributed, when testing the whole data set (Kolmogorov–Smirnov test), linear relationships between SI L/SI M-ratio, SI L, R2*, and log-transformed biopsy LIC values were investigated by Spearman Rank test. Regression lines (calibration curves) were calculated by Passing-Bablok regression analysis. The Cusum test for linearity [67] was used to test the applicability of the Passing-Bablok method [68].

The data were generated by investigating the same pig several times during dextran-iron loading to obtain a wide range of LIC values, while keeping the number of pigs acceptably low, for animal ethical and economic reasons. The drawback of this strategy was the potential generation of nesting effects from each pig. Therefore, when searching for the best calibration curve, with the lowest variance, describing the relationship between SI L/SI M-ratios, SI L values, and biopsy LIC values at different TRs, and when studying the usefulness of the muscle reference, we used linear mixed-effects (LME) models [69]. Two LME models were considered with log-LIC as the dependent variable, pig-id as a random effect, and an interaction term between TR and SI L/SI M-ratio or SI L as independent variables. For each model, a variance function was estimated, where the residual variance is proportional to an exponential function of an interaction between a TR-dependent parameter and the SI L/SI M-ratio and SI L values, respectively. Model control was performed by inspecting the standardized residuals as a function of the predicted values. Pointwise standard deviations, as a function of the SI L/SI M-ratios and SI L values, were plotted for each of the models.

*P* values < 0.05 were used to define a significant difference.

The LME models were estimated using R, version 4.03, and the R-package nlme [69]. Bland–Altman analysis, Passing-Bablok regression analysis, Kolmogorov–Smirnov test, Cusum test, and Spearman Rank test were performed by use of XLSTAT, Live Science for MAC, version 2020. 4.1, Addinsoft. Other Statistical analyses and data handling were performed using StatView version 5.0 for MAC 1992–1998 SAS Institute Inc.

## Results

### Relationship of LIC to SIR and R2*

**Fig. 4:**
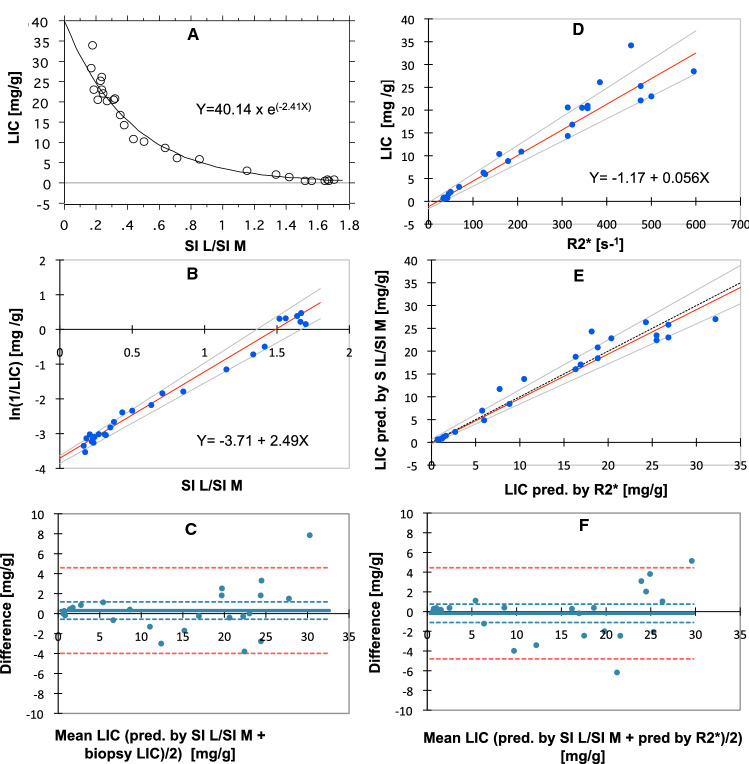
Estimation of LIC by the SIR 25/560 method and by R2* method. **A** and **B** display the relationship of the SI L/SI M-ratio to the biopsy LIC. In **A** data are fitted by an exponential model and in **B** by a linear model after log-transformation. **D** displays the relationship of R2* to biopsy LIC. Regression lines are given in red with 95% CI limits in gray. **C** displays an analysis of agreement between biopsy LIC and LIC estimated by the SIR 25/560 method and **F** between the SIR 25/560 and the R2* method (Bland Altman analysis). The full, bold lines represent bias. Broken, blue lines represent 95% CI for bias and 95% CI for method agreement (broken, red lines). **E** shows the agreement between the SIR 25/560 and the R2* method investigated by linear regression. The regression line is given with 95% CI limits (gray lines) and line of identity (broken, black line). All linear regression analyses by the Passing-Bablok method. *N* = 27

First, we investigated the relationship of biopsy LIC to the SI L/SI M-ratio, using TE = 25 ms (TR = 560 ms), in the following called the SIR 25/560 method, according to the setup, we still use at our Department of Radiology. In total, 27 biopsy LIC measurements and corresponding SIR values were available for image analysis from 13 mini pigs. The numbers of examinations, contributed by each pig, and iron-loading details are displayed in Fig. 2D. Biopsy LIC-values ranged from 0.62 to 34.2 mg/g. Higher biopsy LIC values were excluded because their corresponding SI L levels equaled background levels. The results are displayed in Fig. 4A, showing a curvilinear relationship between the SI L/SI M-ratios and the biopsy LIC values. Data fitted an exponential model (Rho − 0.96, *p* < 0.001, Spearman Rank correlation on a log-linear plot). The equation is given in the figure. The assessed calibration line is displayed in a reciprocal form in Fig. 4B. Its equation is given in the figure. The 95% CI was − 3.86 to − 3.66 for the slope and 2.31 to 2.68 for the intercept.

Then we studied the agreement between LIC estimates, predicted by the SIR 25/560 method, and the corresponding biopsy LIC measurements by the Bland–Altman analysis. Results are given in Fig. 4C and Table 3.

**Table 3:** Investigation of agreement between MRI methods and biopsy LIC by Bland–Altman analysis

Compared methods	95% CI of LoA	
	mg/g	%
SIR contra biopsy	− 4.0 to 4.60	− 29.90 to 39.94
R2* contra biopsy	− 5.27 to 6.21	− 41.29 to 39.79
SIR contra R2*	− 4.79 to 4.45	− 34.00 to 42.27

*LoA* limits of agreement, *CI *confidence interval

The relationship of R2* to biopsy LIC values is displayed in Fig. 4D, showing the data fitted by a linear model (Rho 0.964, *p* < 0.001). The 95% CIs were 0.05 to 0.06 for the slope and − 1.62 to − 0.30 for the intercept. Results of agreement analysis are given in Table 3.

Then we studied the method agreement between LIC estimates, predicted by the SIR 25/560 method and by the R2* method, by the Bland–Altman analysis (Fig. 4F; Table 3). The analysis demonstrated a small bias of − 0.17 mg/g (4.12%) with a 95% CI from − 1.11 to 0.76 mg/g (− 3.58 to 11.82%). Investigation of the relation between LIC estimates, obtained by the SIR 25/560 method, and by the R2* method, revealed a linear relationship (Fig. 4E) with a slope of 0.974 (95% CI 0.88 to 1.09), not different from 1, indicating a proportionality slope, and an intercept of − 0.131 (95% CI − 0.37 to 0.67), not significantly different from zero, indicating no significant systematic difference between the methods.

### Transferability of the calibration curve from the pig model to patients

We compared LIC values, predicted by use of the porcine calibration curve, assessed by the SIR 25/560 method, from SI L/SI M-ratios, assessed in the pigs, with LIC estimates, obtained by translation of the same SI L/SI M-ratios to LIC estimates, by use of the human calibration, still in use at our Department of Radiology for our SIR method. The same MRI machine and scanner software package was used for the patients and the pigs and the applied SE sequence, coil configuration, and imaging parameters were also identical, except a different TR (560 ms instead of 684 ms).

**Fig. 5:**
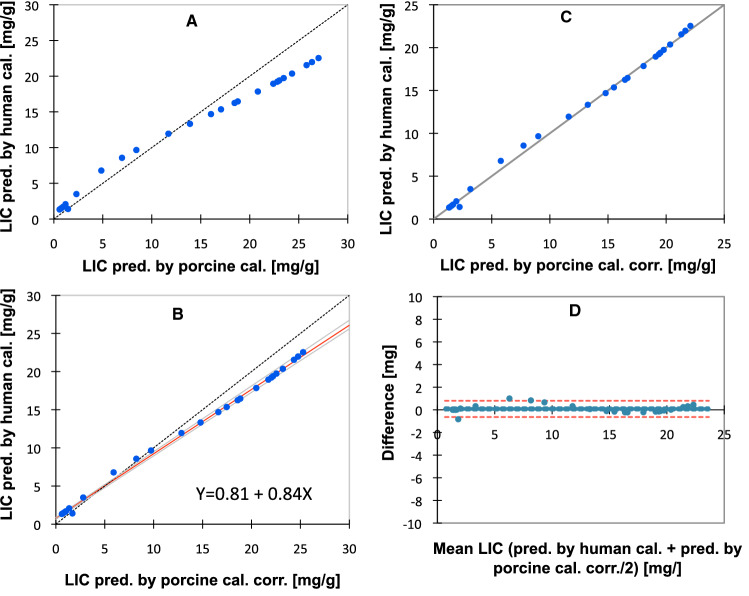
Comparison of LIC estimates predicted by the porcine and a human calibration curve. **A** Displays the relationship between both predicted LIC estimates. The broken line is the line of identity. In **B** porcine SI L/SI M-ratios were corrected for the difference in TR at image acquisition. Linear regression by the Passing-Bablok method, given with 95% CI limits (gray). The regression line is in red. The broken, black line is the line of identity. **C** shows agreement between predicted LIC estimates with the line of identity, calculated by the human and porcine calibration, after correction of the porcine LIC estimates for the proportional and systematic differences, observed in **B**, by use of the correction function displayed in **B**. **D** Displays the method agreement (Bland–Altman analysis). Data are the same as in **C**. The full, bold line represents bias. Broken lines represent 95% CI for bias (blue), and 95% CI for method agreement (red). N = 27

Figure 5A displays the relationship between LIC estimates, obtained by this human calibration (TR = 684 ms), and by the porcine calibration. The graph is not linear, primarily, because a different TR had been used for image acquisition in the pigs. To adjust the SI L/SI M-ratios for the difference in TR, we used two correction functions, implemented in the software package of our SIR method used for humans. For details, see the *Materials and Methods*. After correction of the SI L/SI M-ratios, the LIC values, predicted by the human calibration, were linearly correlated to those, predicted by the porcine calibration, (Fig. 5B). However, the calculated regression equation still indicated a systematic and a proportional difference between both calibrations: The slope coefficient of 0.84 (95% CI 0.32 to 0.86) was significantly different from one, and the intercept of 0.81 (95% CI 0.63 to 0.88) was significantly different from zero. Subsequent use of the regression equation from Fig. 5B as a correction function, led to an identity relationship between both LIC estimates (intercept 0.001 with 95% CI between − 0.20 and 0.08, and a slope of 1.0 with 95% CI from 0.98 to 1.02). Then, the Bland–Altman analysis showed a negligible bias of 0.08 mg/g with 95% CI from − 0.07 to 0.22 mg/g (0.12% with 95% CI from − 3.87% to 4.12%) and 95% LOAs between − 0.64 to 0.80 mg/g (− 19.67 and 19.92%).

### Relation of biopsy LIC to SI L and the SI L/SI M-ratio when decreasing TE:

**Fig. 6:**
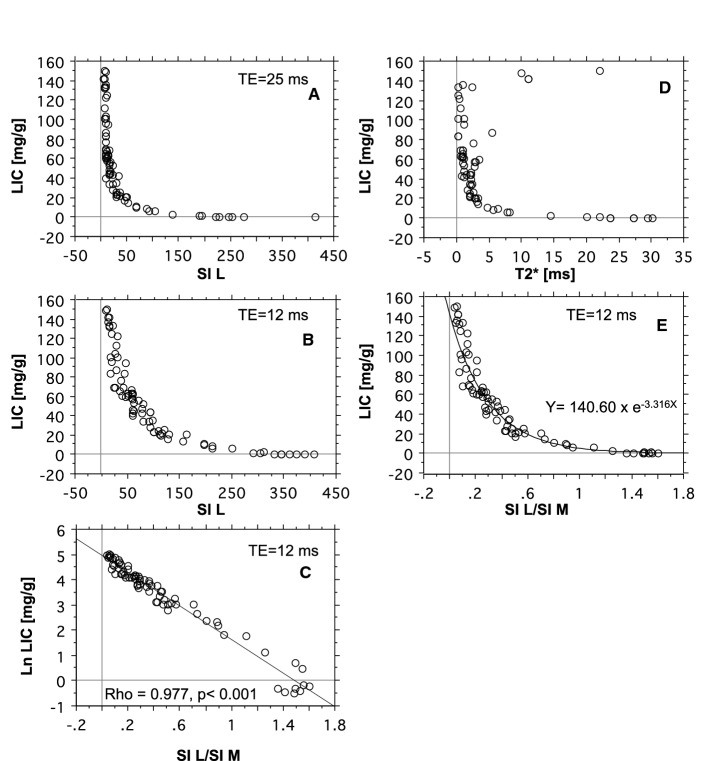
Relation of biopsy LIC to SI L and SI L/SI M-ratio at different TRs and to T2*. The relationship for the SIR 25/560 method is given in **A** and **B** after decreasing TE from 25 to 12 ms. **D** Illustrates the relationship between T2* and biopsy LIC. **C**, **E** Illustrate the relationship between biopsy LIC and SI L/SI M-ratio at TE = 12 ms, in panel E fitted by an exponential model and in **C** by a linear model after log-transformation of biopsy LIC values (Passing-Bablok method). *N* = 74

Seventy-four MRI examinations were available for studying these relations. LIC values spanned from normal up to 150 mg/g in the liver biopsies from the most heavily iron-loaded pigs. The numbers of examinations, contributed by each pig, and further iron-loading details are displayed in Fig. 2B. Figures 6A, B show how the reduction of TE from 25 to 12 ms, while keeping TR at 560 ms, influences SI L. As expected, the signal decays later at increasing LIC values using TE 12 ms, compared to 25 ms. Thus, SI L values below 25 were already seen around biopsy LIC values of 30 mg/g using TE = 25 ms, while, when applying TE = 12 ms, SI L still was around 120. SI L values below 25 were first seen at around 100 mg/g.

Figure 6D illustrates the relation between biopsy LIC and hepatic T2*. The relationship has a similar shape as for the SIR 25/560 method: T2* values approached zero when LIC values exceeded around 35 to 40 mg/g, due to lack of signal intensity.

Figure 6E shows how the SI L/SI M-ratios are related to biopsy LIC values, at TE = 12. Data fitted an exponential model. Moreover, the measuring range of LIC values is markedly increased, compared to the SIR 25/560 method and the R2* method. LIC values within the normal range and up to around 115 mg/g are fitted by the exponential model. Log transformation of the LIC values resulted in a close, linear relationship (Rho = 0.98, *p* < 0.001), (Fig. 6C). Despite a high Rho value, at S IL/S IM ratios > 0.4, corresponding to ln LIC < 3.5 (equals < 34 mg/g), data were not fitted very well by the model.

### Influence of TR on the relationship between SI L, SI L/SI M-ratio, and chemical LIC

We examined whether a change of TR might improve the degree of correlation between biopsy LIC values and SI L values and SI L/SI M ratios, especially within the LIC range below 34 mg/g. MR images were, therefore, acquired at six different TRs (300, 400, 600, 800, 1000, and 1200 ms). Eighty-one MRI examinations (54 at TR = 300 ms), were performed in 13 pigs. As the exponential model did not fit LIC values of more than around 115 mg/g very well, we decided to exclude values ≥ 115 mg/g (11 examinations with LIC values from 117.5 to 143 mg/g), leaving 70 examinations (43 examinations at TR = 300 ms). The numbers of examinations, contributed by each pig, and further iron-loading details are displayed in Fig. 2C, E.

**Fig. 7:**
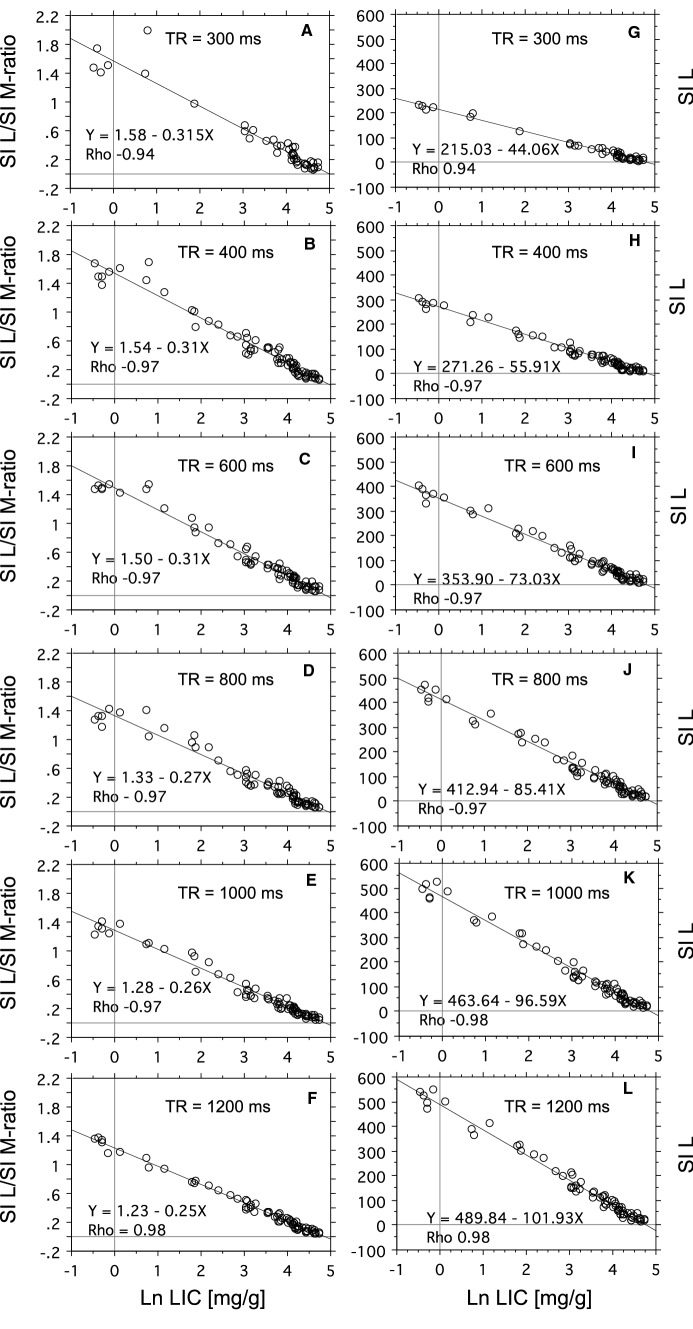
Relationship of biopsy LIC to SI L and SI L/SI M-ratio at different TRs. The relationship was studied at six different TRs, as indicated in each panel. TE was 12 ms. **A**–**F** Displays the relationship for SI L/SI M-ratio and panel G to L for SI L. Linear regression analyses by the Passing-Bablok method. Correlation analysis by Spearman Rank test. *N* = 43 at TR = 300 ms and N = 70 at longer TRs.

Figure 7A–F shows how the SI L/SI M-ratio is related to the log-transformed LIC when using different TRs. The strength of the relationship was studied by Spearman’s Rho. According to this test, the strength of the relationship was not influenced by the selection of TR between 400 and 1200 ms (Rho − 0.97 to 0.98), except at 300 ms, showing a weaker relationship (Rho − 0.94).

**Fig. 8:**
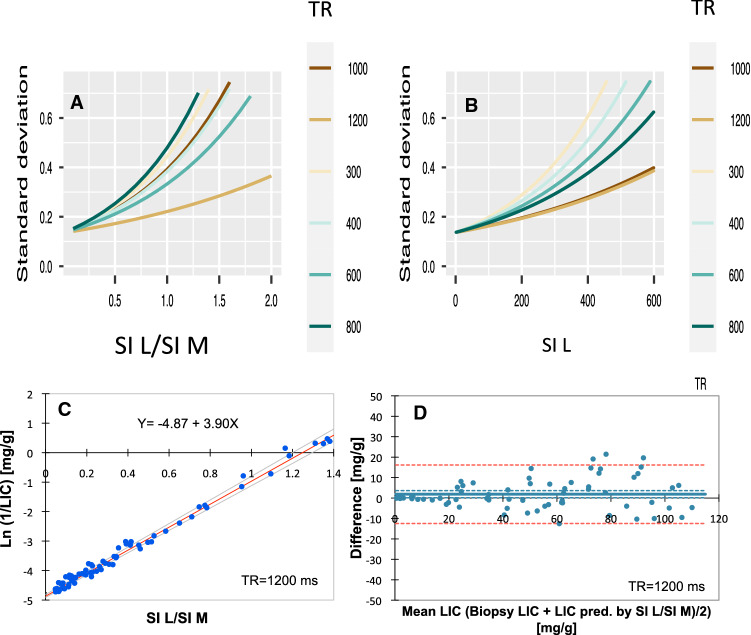
Variance around calibration curves, depending on the applied TR and use of the tissue reference, investigated by LME modeling. The analysis is based on the same data as Fig. 7. Pointwise standard deviations as a function of the SI L/SI M-ratios (**A**) and SI L values (**B**) are plotted for each model. For more details, see the *Statistics.* **C** Displays the calibration curve with the lowest variance, using TR = 1200 ms and the tissue reference (SIR12/1200 method). The regression line is in red (assessed by Passing-Bablok analysis), given with 95% CI limits (gray lines). **D** shows the agreement between biopsy LIC values and those predicted by the calibration curve from **C** (Bland–Altman analysis). The full, bold line represents the bias. Blue, broken lines represent 95% CI for bias, and red, broken lines the 95% CI for method agreement

As Spearman’s Rho is a measure of correlation, but not a measure of linearity, we also used LME to find the TR value leading to the calibration line with the lowest variance (for details see the statistics). The results are displayed in Fig. 8A. The LME model identified TR = 1200 ms as the calibration curve with the lowest variance, increasing exponentially from around 0.1 SD at the highest to around 0.4 SD at the lowest LIC values. Moreover, at high LIC values ≥ 34 mg/g, corresponding to SI L/SI M-ratios < 0.4, the model indicated small differences in variance between calibration curves at different TRs.

### Significance of the skeletal muscle as reference tissue

To investigate the usefulness of the skeletal muscle as reference tissue, we investigated, if SI L/SI M (Fig. 7A–F) or SI L (Fig. 7G–L) was more closely related to Ln biopsy LIC, by comparing Spearman’s Rho. We found that the use of the muscle reference had no improving impact on the degree of correlation at any TR, as all Rho values were the same when using the reference or not. However, an inspection of panels F and L of Fig. 7 suggested a better linear model fit when using the reference at lower LIC levels (SI L/SI M ratios ≥ 0.4) when using TR = 1200 ms. Looking at the LME models (Fig. 8A, B) confirms this effect of the use of the reference within the low LIC range, by demonstrating a slightly less steep variance curve at TR = 1200 ms. Moreover, the models also demonstrated a much steeper increase of the variance curves at lower TRs when using the reference.

As the model indicated different strengths of the relationship between biopsy LIC and MRI-estimated LIC at high and low LIC range, we studied the relationship within the range < 34 mg/g and ≥ 34 to < 115 mg/g by calculating R^2^ (data were normally distributed in the two LIC subsets). The results are shown in Table 4. Overall, the use of the reference slightly increased *R*^2^ at TR = 1200 ms within both LIC ranges, while at lower TRs, the reference had no effect within the high range, but within the low range, all *R*^2^ values were lower when using the reference.

**Table 4:** Relationship between biopsy LIC and MRI estimated-LIC within low and high LIC range at different repetition times (TR)

LIC range	Method	TR (ms)					
		300	400	600	800	1000	1200
Low	SI L/SI M-ratio	0.81	0.91	0.94	0.90	0.95	0.99
Low	SI L	0.99	0.97	0.96	0.96	0.97	0.97
High	SI L/SI M-ratio	0.70	0.82	0.79	0.78	0.83	0.85
High	SI L	0.71	0.81	0.78	0.78	0.82	0.83

Strength of the relationship was studied by calculating *R*^2^. Low LIC range: ≤ 34 mg/g; high LIC range: > 34 to < 115 mg/g.

Finally, we studied the agreement between biopsy LICs and LIC estimates, calculated by using the best calibration curve, with the lowest variance, covering the whole LIC range (from normal to < 115 mg/g), assessed by the use of TR = 1200 ms and TE = 12 ms, by Bland–Altman analysis (Fig. 8D). The 95% LoAs between biopsy LIC and LIC estimates were from − 12.47 to 16.18 mg/g (− 24.58 to 32.33%) and there was only a small bias of 1.86 mg/g (3.88%) with a 95% CI from 0.11 to 3.60 mg/g (0.42% to 7.34%). The regression equation of this calibration curve is given in Fig. 8C.

## Discussion

Today, most MRI methods for estimation of liver iron rely on the T2 [42] or T2* effect [35–40, 43, 44], because the relaxation enhancement effect of iron on T2 and T2* is much greater than on T1 [70]. In the present study, we compared our T1-weighted SIR 25/560 method with a T2* method. Agreement between biopsy and MRI-estimated LIC values was similar as well as the upper limit of the measuring range around 35 mg/g. We optimized our SIR method to extend its limited measuring range. For these investigations, we used a mini-pig model of iron overload, because this enabled the calibration of the method over a wide LIC range.

### Comparison of LIC measuring range of our method with other methods

Many studies, which have investigated the relationship between MRI-derived LIC estimates and the chemical LIC, include one or a few patients with LIC levels exceeding 40 mg/g dry weight [25, 34, 36, 40, 71, 72]. These very high LIC values exceed the upper measurable range of 35 to 40 mg of the R2* method of Wood et al.[36], are equal to or exceed the highest LIC levels estimated by the FerriScan® method[42], and significantly exceed the upper limit of the measuring range of our SIR 25/560 method of 30–35 mg/g [47].

In the present study, we were able to extend the upper LIC limit of the measuring range of our method to around 115 mg/g. Such a high upper limit has not been reported before, and it is unlikely that patients with higher LIC levels will be encountered in clinical practice. The extension of the measuring range was achieved by studying the effect of changing of TE and TR on SI L and the SIR of liver tissue in iron-loaded mini pigs within a wide range of iron overload, including very high LIC values. As we had expected, the reduction of TE from 25 to 12 ms indeed caused a later decay of the SI L at high LIC values, extending the upper limit of the measuring range, but also led to a lower precision at low LIC values below < 34 mg/g. These observations suggest a lower sensitivity to the iron of the applied T1-weighted sequence after TE reduction that appeared to be canceled within the low LIC range by choosing a longer TR.

An extension of the measuring range of the R2* method is also under investigation: Very recently a new, promising approach has been presented, based on the use of ultrashort echo time images (< 1 ms) [73, 74]. Operating at 3 Tesla, the technique could reliably estimate high liver iron concentrations up to 50 mg/g dry weight [74]. It was concluded that further improvement to the protocol and fitting approaches may allow measuring low and moderate LIC with the same accuracy demonstrated at high iron concentrations. [74].

### Comparison of agreement between LIC assessed by biopsy and by MRI:

Using the SIR 12/1200 method, the 95% LoAs between biopsy LIC values and those, estimated by the SI L/SI M ratios, were − 24.58 to 32.33%. This range of LoAs is smaller than those published for the T2* method (− 46 to 44%) [36] and the FerriScan^®^ method (− 56 to 50%) [42]. The agreement was not investigated in the study of Gandon et al. [43]. There are several possible explanations for the better agreement as follows:

One likely explanation is that mini pigs do not develop liver fibrosis despite very severe, long-standing, hepatic iron loading with weekly dextran-iron injections [59]. In patients with iron-loading anemias and transfusional iron overload, liver fibrosis is a well-known complication [7–9]. Liver fibrosis has an important impact on the coefficient of variation (CV) of multiple measurements of the iron concentration in percutaneous needle biopsy from the liver. Thus, in non-diseased livers, CV values of 19% have been reported [75] and for cirrhotic livers, the CV has been reported to be as high as 41% [76]. The sampling error of the liver biopsy is regarded to be the major contributor to the span of LoA for the FerriScan^®^, R2-based method [42] and also for the T2* method [36]. The underlying reason for the increased sampling error of biopsy LIC measurements with increasing fibrosis stages is the lack of iron in fibrous tissue and the spatial inhomogeneity of fibrosis within the liver [77].

A further potential explanation for the better agreement is that the lack of hepatic fibrosis in the mini pigs excludes a potential interference of fibrosis on the relaxation processes in the liver tissue in the present study. Thus, it has been shown that the presence of fibrosis within the liver may significantly increase the relaxation times T1 [78, 79] and T2 [79] of the liver tissue. A similar interfering effect has also been claimed for R2* [80]. However, R2* was not found to be significantly related to low-grade fibrosis in a study of patients with transfusional iron overload [37].

Another likely reason is the larger sample weight of the percutaneous liver biopsy in the present study. Thus, it has been shown that the variability of LIC measurements increases with smaller biopsy samples less than 1 mg dry weight [81]. The mean dry weight of the liver needle biopsies in the present study was 2.52 ± 1.11 mg in 81 samples. Only five biopsies weighed less than 1 mg. In the study of Wood et al*.* [36], the range of sample weight, measured in four patients, was from 5.1 to 6.3 mg wet weight, equaling 1.5 to 1.8 mg dry weight. In the study of St Pierre et al. [42], all samples had a dry weight of more than 0.4 mg.

Moreover, the later decay of SI L at high LIC may have improved the agreement between LIC estimates by MRI and biopsy LIC levels, especially at high LIC levels. Also, the fact that liver biopsy and MRI always were performed on the same day, and the lack of motion artifacts due to the anesthesia of the pig, may all have contributed to a better agreement.

### The usefulness of the tissue reference

The most important drawback of the SIR method, in contrast to the relaxometry methods, is the need for comparison of SIL to a reference tissue that does not accumulate iron, because the absolute signal intensity of a tissue, measured by MRI, is arbitrary and depends on imaging parameters [32]. Relaxometry methods do not need a tissue reference, because they model the signal intensity of a single tissue (e.g. liver) across multiple TEs, enabling the calculation of T2 and T2* [32]. The SIR method, used in the present study, applies skeletal muscle as the reference tissue, which has not been shown to accumulate iron despite heavy iron loading in mini pigs [59]. However, there is some indication of lack of inter-scanner reproducibility of SIR methods from one study [38] as follows: Alterations of the SIR of liver tissue were noticeable between scanners, which were ascribed to an observed variation of the signal intensity of the paraspinal muscle between different gradients and equipment.

In the present study, we found that the usefulness of the muscle reference depended on the applied TR and the level of LIC. This underlines the significance of selecting the optimal TR for a SIR method. This optimization can easiest be performed in a model of iron overload, supplying subjects with a wide range of different LICs, as it is possible with the mini-pig model used in the present study.

### Transferability of the calibration curve from pig model to patients

Finally, it must be asked if the calibration curve for the SIR 12/1200 method, optimized in the pig model, can directly be used for estimation of LIC in patients with iron overload. Although the pig model has been shown to simulate humans very well regarding anatomy, physiological and metabolic processes [82, 83], we found a proportional difference of around 16% when comparing the porcine with the human calibration curve for the SIR TE 25/560 method. The most likely explanation for this difference is the different processing of the liver tissue from the needle biopsy used for the determination of LIC. In the present study, desiccated tissue from fresh liver specimens was used, while for the human calibration, deparaffinized liver tissue samples were used. The paraffin embedding technique has been shown to overestimate the LIC [84, 85], most pronounced in the study of Butensky [84], demonstrating an overestimation of 23%. Paraffin embedding of tissue samples includes washing in xylene, which dissolves lipids, reduces dry weight, and increases the iron-to-weight ratio compared to fresh samples [84]. This likely explanation of the difference suggests that the porcine calibration curve for the SIR 12/1200 method is also useful for the estimation of LIC in humans.

However, before the porcine calibration can be used for translation of SIR values to LIC values in humans, a study is necessary, comparing parallel LIC estimates, predicted from SIR values measured by the SIR 25/560 method and by the SIR 12/1200 method in humans with iron overload, and if possible, with parallel LIC measurements in desiccated fresh liver biopsy specimens from the patients. The lack of these data represents the most important limitation of the present study. Another limitation is that the SIR 12/1200 method has not yet been validated across different scanner types. This is relevant because limited inter-scanner reproducibility of SIR methods has been reported [38].

## Conclusion

Various MR imaging techniques, each with their respective advantage and limitations, have been developed for the non-invasive estimation of LIC. The advantage of the presented SIR12/1200 method is the much wider measuring range, up to LIC values well above 100 mg/g, and a good agreement between LIC values assessed by biopsy and by MRI, if appropriate scanning parameters are used. The extended measuring range is likely caused by lesser sensitivity of the SE sequence to iron, due to shorter TE, leading to later signal loss at high LIC. The advantages of the method will probably enable a proper non-invasive evaluation of a wide range of patients with iron overload, including the most severe cases.

## Abbreviations

CI: Confidence interval
ICP-OES: Inductively coupled plasma-optical emission spectroscopy
GRE: Gradient echo
LIC: Liver iron concentration
LME: Linear mixed effect
LoA: Limits of agreement
MRI: Magnetic resonance imaging
ROI: Region of interest
SE: Spin echo
SI: Signal intensity
SI L: Signal intensity of liver tissue
SI M: Signal intensity of muscle tissue
SIR: Signal intensity ratio
TE: Echo time
TR: Repetition time
